# High-fructose and high-fat diet-induced disorders in rats: impact on diabetes risk, hepatic and vascular complications

**DOI:** 10.1186/s12986-016-0074-1

**Published:** 2016-02-25

**Authors:** Iona Lozano, Remmelt Van der Werf, William Bietiger, Elodie Seyfritz, Claude Peronet, Michel Pinget, Nathalie Jeandidier, Elisa Maillard, Eric Marchioni, Séverine Sigrist, Stéphanie Dal

**Affiliations:** UMR DIATHEC, EA 7294, Centre Européen d’Etude du Diabète, Université de Strasbourg, Fédération de Médecine Translationnelle de Strasbourg, Bld René Leriche, 67200 Strasbourg, France; Equipe de Chimie Analytique des Molécules BioActives, IPHC-LC4, UMR 7178, Faculté de Pharmacie, Ilkirch, France; Structure d’Endocrinologie, Diabète, Nutrition et Addictologie, Pôle NUDE, Hôpitaux Universitaires de Strasbourg, (HUS), 67000 Strasbourg, France

**Keywords:** High-fat high-fructose diet, Metabolic syndrome, Type 2 diabetes, Oxidative stress, Hepatic complications, Vascular complications, Endothelial dysfunction

## Abstract

**Background:**

As a result of the increased consumption of sugar-rich and fatty-products, and the increase in preference for such products, metabolic disorders are becoming more common at a younger age. Fructose is particularly used in prepared foods and carbonated beverages. We investigated the impact of regular consumption of fructose, in combination or not with fatty food, on the onset of metabolic syndrome and type 2 diabetes (T2D). We evaluated the metabolic, oxidative, and functional effects on the liver and blood vessels, both related to diabetes complications.

**Methods:**

High-fat diet (HFD), high-fructose beverages (HF) or both (HFHF) were compared to rats fed with normal diet (ND) for 8 months to induce T2D and its metabolic, oxidative, and functional complications. Metabolic control was determined by measuring body weight, fasting blood glucose, C-peptide, HOMA2-IR, leptin, and cholesterol; oxidative parameters were studied by lipid peroxidation and total antioxidant capacity in plasma and the use of ROS labelling on tissue. Histological analysis was performed on the liver and endothelial function was performed in main mesenteric artery using organ-baths.

**Results:**

After 2 months, HFHF and HFD increased body weight, leptin, HOMA2-IR associated to steatosis, oxidative stress in plasma and tissues, whereas HF had only a transient increase of leptin and c-peptide. Only HFHF induced fasting hyperglycaemia after 6 months and persistent hyperinsulinaemia and fasting hyperglycaemia with complicated steatosis (inflammation and fibrosis) after 8 months. HFHF and HFD induced endothelial dysfunction at 8 months of diet.

**Conclusions:**

Six months, high fat and high carbohydrate induced T2D with widespread tissues effects. We demonstrated the role of oxidative stress in pathogenesis as well as in complications (hepatic and vascular), reinforcing interest in the use of antioxidants in the prevention and treatment of metabolic diseases, including T2D.

## Background

Food and beverages rich in energy, fat, and/or sugar are now commonly consumed in modern societies [1]. In addition to genetic predisposition [2], physical inactivity [1], and perinatal environment [1, 3], such diets are recognized as major causes of the obesogenic environment in humans [1]. The consumption of large amounts of added sugar, a prominent source of low-nutrient calories in processed or prepared foods and caloric beverages (i.e. soft drinks, colas) is a relatively new phenomenon [4]. In the mid-19^th^ century, these sweeteners became widely available and their consumption began to increase dramatically [5]. Fructose is used commercially as a sweetening substitute (fructose corn syrup) for glucose or sucrose, in the preparation of desserts, condiments, and carbonated beverages [6]. It has been recently confirmed [7] that the consumption of high amounts of refined carbohydrates in food and beverage increases the risk of dyslipidaemia [8], obesity [4, 6], insulin resistance [9], and heart disease [10]. A recent epidemiological analysis in humans also found an association between diabetes prevalence and sugar availability [11]. Moreover, chronic consumption of a Western diet, characterized by foods rich in sugar and abundant in total and saturated fat, has been suggested to play a role in the development of type 2 diabetes (T2D) [12].

Diabetes is known to produce substantial changes in intracellular metabolism in most tissues, including liver [13]. Insulin resistance and excessive accumulation of lipids is strongly associated with non-alcoholic fatty liver disease (NAFLD), which represents the hepatic manifestation of a systemic impairment of the insulin network [14].

In addition to being a secondary consequence of metabolic syndrome, NAFLD is also in itself a major risk factor for diabetes [15], and also contributes to cardiovascular morbidity and mortality, with a two-fold increase in the risk of death [16]. One of the alterations that characterize NAFLD is hepatic steatosis, associated with obesity, insulin resistance, diabetes mellitus, and metabolic syndrome. Hepatic steatosis is characterized by the presence of hepatic fat accumulation, which, unlike non-alcoholic steatohepatitis (NASH), is not accompanied by ballooning of hepatocytes [17]. NAFLD includes a spectrum of diseases, ranging from simple fatty liver to NASH, which may progress to end-stage liver disease (cirrhosis) and hepatocellular carcinoma, requiring hepatic transplantation. This pathogenesis is multifactorial and includes lipid metabolism alterations, with an aberrant accumulation of triglycerides, mitochondrial dysfunction, inflammation, and oxidative stress (OS) [18].

OS, defined as an impaired balance between free radical production and antioxidant capacity resulting in accumulation of oxidative products [19], is a well-recognized mechanism that plays important roles in many pathological conditions. Several human diseases have been closely associated with OS [20], including aging [21], metabolic syndrome [20], and diabetes [20]. Several studies, which have proposed mechanisms to explain the increased OS in both forms of diabetes, suggest that diabetes is a bipolar process in which, on one hand, there is an increase in generation of reactive oxygen species (ROS), and, on the other hand, a decrease in the levels of plasma antioxidants levels such as vitamin E, vitamin C, lipoic acid, and glutathione [22]. Recent studies have shown that OS induces changes in redox balance resulting in dysregulation of redox biology [19, 20], and plays an important role in liver disease [18]. Moreover, OS has been closely related to cardiovascular diseases [20] linked with diabetes.

Diabetes-related vascular complications are an important pathological issue which lead to the functional deterioration of several organs, and cause micro- and macro-angiopathy [23]. Large clinical studies of both forms of diabetes have demonstrated that hyperglycaemia plays an important role in the pathogenesis of microvascular complications [23]. Moreover, there is considerable evidence demonstrating impairment of endothelium-dependent vasodilatation in cardiovascular diseases. This impairment of microvascular blood flow occurs early in the pathogenesis of T2D, with evidence at the time of diagnosis [24]. Dysfunction of the endothelium is regarded as an important factor in diabetes [24] and has gained increasing attention in the study of vascular disease.

Animal models have contributed greatly to the study of diabetes. Such models allow researchers to control, in vivo, genetic, and environmental factors that may influence the development of the disease and its secondary complications, therefore gaining useful information on its management and treatment in humans. There are many animal models of obesity and T2D [25], some of which show a genetic predisposition to the disease [26], while others may develop the disease spontaneously [27] or in a diet-induced manner [28]. The most commonly used non-genetic rodent models of diabetes are those induced by streptozotocine or alloxan, in addition to diet [29], or models obtained by partial pancreatectomy [25] which leads to insulin deficiency, hyperglycaemia, and ketosis. Although these models are useful for the study of diabetes, they are not representative of diet-induced human metabolic syndrome and T2D. Diet composition has been considered an important factor in the impairment of insulin activity [28]. Our previous study showed that the administration of a high-fat diet (HFD) to rats for 2 months is a fast and easy way to induce metabolic syndrome, associated with metabolic and oxidative disorders, without modulation of glycaemia [30]. However, recent epidemiological studies of sugar consumption and diabetes prevalence [11] suggest that a diet rich in fat as well as sugar is a greater risk factor for these disorders than a diet that is rich in either fats or sugars.

The aim of our study was to determine the impact of sugar on the development of metabolic syndrome and its evolution into T2D, as well as on the development of related secondary complications. We compared the impact of a diet rich in both sugar and fat with that of a sugar-rich diet without the addition of fat. We measured metabolic parameters such as insulin resistance, glucose tolerance, fasting glycaemia, and compared hepatic (steatosis, inflammation) and vascular (endothelial function) complications.

## Methods

### Chemicals

2,2′-Azino-bis-(3-ethylbenzthioazoline-6-sulfonic acid) (ABTS) was purchased from VWR (Fontenay sous Bois, France), amyloglucosidase (AMGD) from Roche Diagnostic (Meylan, France), glucose and phosphate-buffered saline (PBS) from Fisher Scientific (Illkirch, France), eosin, Harris hematoxylin, paraffin, ethanol and toluene from Labonord (Templemars, France). The (+)-6-hydroxy-2,5,7,8-tetramethylchroman-2-carboxylic acid (Trolox) and all other products were purchased from Sigma-Aldrich (St Quentin Fallavier, France).

### Ethics statement

The study was performed in accordance with the “Guide for the Care and Use of Laboratory Animals” published by the US National Institutes of Health (NIH publication No. 85–23, revised 1996), and the present protocol was approved by the local ethics committee (Comité Régional d’Ethique en Matière d’Expérimentation Animale CREMEAS, approval AL/02/11/05/12). All efforts were made to minimize animal suffering and reduce the number of animals used.

### Animals and induction of diabetes

Sixty-five male Wistar rats (8 weeks old; 204 ± 1 g), supplied by Depré (Saint Doulchard, France), were housed in a temperature-controlled room, in a 12-h-light/dark cycle environment with *ad libitum* access to water and food. At the beginning of the study, 5 rats were sacrificed (Ctr-rats, M0). After 2 weeks, the rats (312 ± 2 g) were randomly divided into four groups of 15 rats each. The first group had free access to a standard diet “Normal Diet” (ND) from SAFE (Augy, France), with the following macronutrient composition: 3.1 % fat, 16.1 % protein, 3.9 % fibre, and 5.1 % ash (minerals). The second group “High Fructose” (HF) had the same normal diet, but with an additional 25 % of fructose (Sigma, France) in water. The third group, “High Fat Diet” (HFD), received a purified laboratory hypercaloric rodent diet “WESTERN RD” (SDS, Special Diets Services, Saint Gratien, France) containing 21.4 % fat, 17.5 % protein, 50 % carbohydrate, 3.5 % fibre, and 4.1 % ash. The fourth group, “High Fat High Fructose” (HFHF), had both the enriched diet and fructose in water. Both groups had free access to water. The body weight and calorie intake of each animal was recorded once a week. 5 rats were sacrificed at the beginning of the study (M0), and then 5 rats of all groups were sacrificed at 2 and 8 months (M) after starting administration of each diet.

### Sacrifice

Before anaesthesia, body weight was recorded, capillary glucose levels were measured, and tail vein blood samples were taken to estimate metabolic parameters. After anaesthesia with an intraperitoneal injection of 50 mg/kg pentobarbital (Centravet, France), blood was drawn from the abdominal aorta, and plasma and serum were frozen in liquid nitrogen and stored at −80 °C after centrifugation (4 °C, 2 min, 10,000 × *g*) for later biochemical analysis. Liver tissue was cleaned, weighed and embedded in Tissue-Tek® OCT (Optimal Cutting Temperature compound, Leica Microsystem SAS, Nanterre, France) or directly frozen in liquid nitrogen and stored at −80 °C. The main superior mesenteric artery was excised and bathed in Krebs bicarbonate solution (119 mM NaCl, 4.7 mM KCl, 1.18 mM KH_2_PO_4_, 1.18 mM MgSO_4_, 1.25 mM CaCl_2_, 25 mM NaHCO_3_, and 11 mM d-glucose, pH 7.4, 37 °C) for dissection.

### Biochemical plasmatic analysis

#### Plasmatic metabolic parameters

Glucose tolerance was evaluated by measuring intraperitoneal glucose tolerance (IpGTT) of fasting rats. Capillary glycaemia at baseline and 15, 30, 60, and 120 min after an intraperitoneal (IP)-injection of 2 g/kg glucose (20 % solution) was measured with a glucometer (Accu-Chek Performa®, Roche Diagnostic, France). Blood samples were collected from the tail vein at 0 and 60 min after injection, in order to measure blood glucose (glucose RTU®, Biomérieux, France) and C-peptide levels (Elisa C-peptide kit, Mercodia, Uppsala, Sweden) to evaluate insulin sensitivity. Measuring C-peptide was preferred to measuring insulin for evaluating insulinemia, because it is more stable in blood and is not affected by haemolysis [31]. Results were expressed in g/L for plasma glucose and in pmol/L for plasma C-peptide. Fasting leptin was measured by ELISA (Elisa Leptin kit, Linco Research Inc., St Louis, MO, USA) as An index of fat mass [32]. Plasmatic cholesterol was quantified by a colorimetric method Cholesterol RTU™ (BioMérieux, Lyon, France) using a cholesterol calibrator. Insulin resistance was evaluated using the homeostasis model assessment (HOMA2). HOMA2-IR was calculated for fasting plasma glucose and fasting C-peptide using the HOMA2 model calculator (http://www.dtu.ox.ac.uk/homacalculator). All parameters were measured once a month.

#### Plasmatic inflammatory and oxidative parameters

TNFα was assessed on plasma according to the manufacturer’s instructions (Rat TNF-α ELISA Kit, Millipore, Fontenay sous Bois, France). Total antioxidant capacity (TAOC) with the radical cation ABTS^•+^ was performed by a trolox equivalent antioxidant capacity method as previously described [30]. Lipid peroxidation as a consequence of OS was estimated by measuring TBARS using a kit (OxiSelect™ TBARS Assay Kit-MDA Quantitation, Cell Biolabs Inc., San Diego, CA, USA) according to the manufacturer’s instructions, and expressed in μmol/L TBARS.

### Histological and functional studies

#### Morphological analysis and immunohistochemistry

The degree of hepatic histological changes was assessed by eosin/hematoxylin coloration, Oil Red O (steatosis), and Masson’s Trichrome (fibrosis) staining on 10-μm cryosections fixed with 4 % paraformaldehyde. Steatosis was evaluated according to the standard Kleiner Classification [33] of grading and staging. Degree of steatosis was scored as the percentage of hepatocytes per lipid droplet: 0 (less than 5 %), 1 (between 5 and 33 %), 2 (between 33 and 66 %) and 3 (higher than 66 %), complicated or not by fibrosis.

#### *In situ* liver macrophages

As previously described by Dal S et al. [34] frozen-embedded liver sections (10 μm) were fixed and incubated with rabbit anti-Iba-1 (Rat, 1:1000, Wako Chemicals GmbH, Germany). Macrophage density was expressed as the percentage of brown pixels per field in comparison to control values (100 %). Six slides were prepared for each animal, and five fields were analysed per slide at a magnification of × 20.

#### Hepatic triglycerides and glycogen quantification

Extraction of hepatic triglyceride content was performed on piece of fresh liver (100 mg) mixed with a high-speed homogeniser (Polytron PT MR2100, Kinematica AG, Luzern, Switzerland) in a chloroform and methanol buffer (CHCl_3_/Methanol/H_2_O, v/v: 2/1/0,6), and centrifuged (1000 × *g*, 10 min, ambient temperature). The clot was mixed with a fresh solution of chloroform-Triton (X100, 2 %), evaporated (55 °C), and diluted in milli-Q water. Triglycerides were determined using the Triglycerides Quantification Kit (Abcam, Paris, France) according to the manufacturer’s instructions. Samples were measured at 550 nm and concentrations were expressed in nmol/mg of liver.

Hepatic glycogen content extraction was performed on piece of fresh liver (100 mg) according to the method described previously [34] and expressed in mg of glycogen/mg of liver.

#### Tissue oxidative stress

The oxidative fluorescent dye dihydroethidine (DHE) was used to evaluate *in situ* formation of ROS according to a method described by Dal-Ros et al. [34]. Unfixed liver and mesenteric artery were cut into 10-μm-thick sections, treated with DHE (2.5 μM), and incubated in a light-protected humidified chamber at 37 °C for 30 min. The level of ROS was determined using microscopy and whole fluorescence of tissue was quantified with the microscope assistant (NIS-Elements BR, Nikon, France), and expressed as a percentage of that in age-matched ND rats.

As previously described [35], liver tissue (5 mg) from experimental rats was homogenized using NP-40 buffer (NaCl 150 mmol/L, 1.0 % Triton X-100, Tris 50 mmol/L, pH 8) with a protease/phosphatase inhibitor cocktail (Roche Diagnostics, Meylan, France) using an ULTRA-TURRAX. Supernatants were collected and protein contents measured by the Bradford method [36] SOD and catalase activities were performed (50 mg of proteins) according to the manufacturer’s instructions (Superoxide dismutase assay kit and Catalase Assay Kit, Abcam, Paris, France) and expressed respectively in % of inhibition rate and (μmol/L). Lipid peroxidation was estimated by measuring TBARS using a kit (OxiSelect™ TBARS Assay Kit-MDA Quantitation, Cell Biolabs Inc., San Diego, CA, USA) according to the manufacturer’s instructions, and expressed in μmol/L TBARS/mg of proteins.

#### Vascular reactivity studies

Mesenteric artery rings were suspended in organ baths for the determination of changes in isometric tension, as described previously [21]. The NO-mediated component of relaxation was determined in the presence of indomethacin (10 μM) and charybdotoxin (CTX) plus apamin (APA) (100 nM each) to rule out the formation of vasoactive prostanoids and EDHF, respectively. The EDHF-mediated component of relaxation was determined in the presence of indomethacin (10^−5^M) and N^Ѡ^-nitro-l-arginine (L-NA, 10^−4^M) to rule out the formation of vasoactive prostanoids and NO, respectively. Levcromakalim- (an ATP-sensitive K^+^channel opener; 0.1 nM–10 μM) induced relaxations were examined in endothelium-denuded rings of mesenteric artery to test the vascular smooth muscle cells relaxations without EDHF production by endothelial cells.

### Statistical analysis

Values are expressed as means ± SEM, and n indicates the number of rats. Statistical analysis was performed with Student’s t-test for unpaired data or ANOVA followed by Tukey’s protected least-significant difference test, where appropriate (Statistica®, StatSoft, France). *p* < 0.05 was considered to be statistically significant.

## Results

### Metabolic follow-up

**Fig. 1:**
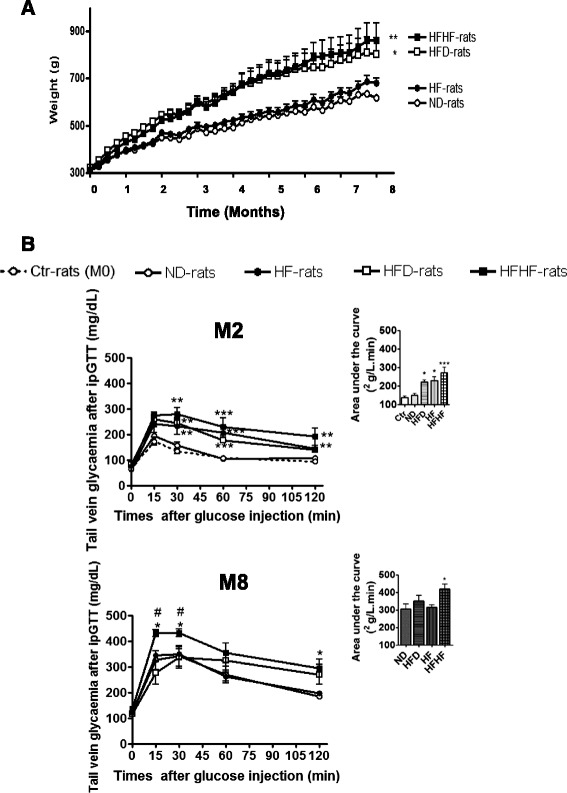
Weight and glucose tolerance measured during the study. **a** Weight during the study for the normal diet (ND, ○), normal diet + fructose (HF, ●), high fat diet (HFD, □) and HFD + fructose (HFHF, ■) groups. * Global significant results *versus* age-matched ND-rats. **b** Evolution of glycaemia and the area under the curves (AUC) during intraperitoneal glucose tolerance (IpGTT) test on fasting rats (0) and 15, 30, 60, 120 min after glucose injection in all groups and at the beginning (Ctr-rats, M0) and after 2 (M2) and 8 (M8) months of diet. Results are shown as mean ± SEM of 5 different experiments. * Significant results *versus* age-matched ND-rats

After 3 weeks of diet, HFHF and HFD induced a significant increase in body weight (*p* < 0.05) maintained until the end of the study, in comparison to HF and ND diet (respectively, 863 ± 72 g; 863 ± 70 g; 680 ± 22 g and 617 ± 9 g) (Fig. 1a). After 2 months, fasting glycaemia (Tables 1, 2 and 3) was higher in HFD and HFHF rats. Moreover, AUC after ipGTT was higher in hypercaloric diet (HF, HFD and HFHF) (respectively: 229 ± 20; 221 ± 12 and 271 ± 30; *p* < 0.01) (Fig. 1b). C-peptide levels in pre- and post-ipGTT were also higher in the 3 groups as compared to those in ND-rats (Tables 1, 2 and 3). After 2 months, HOMA2-IR values were higher than 2.4 in HF, HFD, and HFHF rats, confirming insulin resistance, but only significantly increased in HFD and HFHF rats as compared to ND rats (*p* < 0.01) (Tables 1, 2 and 3). After 4 months, fasting glycaemia was the same in all groups around 104.5 ± 3mg/dL and only HFHF rats shown fasting glycaemia under 126 mg/dL (147 ± 7 mg/dL) after 6 months of diet in comparison to ND, HF and HFD (respectively: 103 ± 2; 114 ± 3 and 105 ± 2 mg/dL) (data not shown).

**Table 1:** Evolution of metabolic parameters during the study

M0	Ctr
Weight (g)	300 ± 6
Fasting Leptin (ng/mL)	3.5 ± 0.4
Fasting glycaemia (mg/dL)	83 ± 3
Peptide-C (pmol/L) Fasting	860.5 ± 56.8
Non-fasting	1283.6 ± 115.3
Insulin resistance (HOMA2-IR)	1.22 ± 0.08
Total cholesterol (mM)	2.20 ± 0.49

Effects of diet on body weight and metabolic parameters during the study, at the beginning (M0) of normal diet (ND), normal diet + fructose (HF), high fat diet (HFD) and HFD + fructose (HFHF)

**Table 2:** Evolution of metabolic parameters during the study

M2	ND	HF	HFD	HFHF
Weight (g)	442 ± 610	462 ± 8	543 ± 14*	553 ± 14*
Fasting Leptin (ng/mL)	8.19 ± 0.44	10.84 ± 0.75**	20.86 ± 0.79***	17.05 ± 1.68*** ^**a**^
Fasting glycaemia (mg/dL)	89 ± 4	102 ± 6	128 ± 6***	112 ± 6**
Peptide-C (pmol/L) Fasting	1027.8 ± 71.4	2340.2 ± 73.7***	1404.5 ± 73.7***	2282.6 ± 187.9***
Non-fasting	1974.5 ± 163.8	5222.9 ± 374.1***	3578.2 ± 256***	4531.3 ± 432.8***
Insulin resistance (HOMA2-IR)	2.20 ± 0.15	3.35 ± 0.33	6.59 ± 0.67***	5.36 ± 0.79*
Total cholesterol (mM)	2.92 ± 0.41	4.0 ± 0.80	3.44 ± 0.48	5.03 ± 0.73*

Effects of diet on body weight and metabolic parameters during the study, after 2 (M2) of normal diet (ND), normal diet + fructose (HF), high fat diet (HFD) and HFD + fructose (HFHF). In bold significant difference with age-matched ND rats and ^a^between HFD- and HFHF-rats. *p<0.05, **p<0.01, ***p<0.001

**Table 3:** Evolution of metabolic parameters during the study

M8	ND	HF	HFD	HFHF
Weight (g)	617 ± 9	680 ± 22	863 ± 70***	863 ± 72***
Fasting Leptin (ng/mL)	22.41 ± 1.48	26.13 ± 1.45	91.17 ± 13.8**	124.11 ± 5.09** ^**b**^
Fasting glycaemia (mg/dL)	124 ± 5	122 ± 4	116 ± 2	137 ± 2* ^**b**^
Peptide-C (pmol/L) Fasting	1871.8 ± 259	2060.7 ± 180.0	2730 ± 365.1	3937.3 ± 4953.1*
Non-fasting	3534.8 ± 185.8	6081.3 ± 987.2	6615 ± 828.3*	12411.7 ± 2072.4** ^a^
Insulin resistance (HOMA2-IR)	4.53 ± 0.62	4.97 ± 0.44	5.7 ± 0.82	9.78 ± 1.24***
Total cholesterol (mM)	1.76 ± 0.24	2.44 ± 0.51	3.0 ± 0.59	4.23 ± 0.47*

Effects of diet on body weight and metabolic parameters during the study, after 8 months (M8) of normal diet (ND), normal diet + fructose (HF), high fat diet (HFD) and HFD + fructose (HFHF). In bold significant difference with * vs. age-matched ND rats and ^a, b^between HFD- and HFHF-rats. *p<0.05; **p<0.01; ***p<0.001 and ^a^ *p*<0.05 and ^b^ *p*<0.001

After 8 months, HFHF maintained all metabolic parameters disorders such as higher fasting leptinaemia, fasting glycaemia, fasting and post-ipGTT C-peptide, as indicated by the AUC (*p* < 0.05) and HOMA2-IR (*p* < 0.001). Glycaemia at 2 h post-ipGTT was increased to greater than 200 mg/dL in HFD rats and associated with a higher level of C-peptide, but without significant increase in fasting glycaemia or HOMA2-IR, as compared to HF or ND rats (Tables 1, 2 and 3). Total cholesterol increased in HFHF rats from 2 months in comparison to ND rats (*p* < 0.05), whereas this effect was not observed for HFD and HF rats (Tables 1, 2 and 3).

### Inflammatory and oxidative follow-up

HFHF and HFD induced respectively an increase of five and four times of the plasmatic TNFα after 8 months (Table 4). Moreover, lipid peroxide levels increased also after 8 months in HFHF and HFD rats (Table 4), whereas HF had no effect on these inflammatory and OS parameters. During the study, the total antioxidant capacity was comparable in all groups, in spite of OS (Table 4).

**Table 4:** Evolution of inflammatory and oxidative parameters during the study

M0	Ctr	M0		Ctr
*Plasmatic parameters*		*Hepatic parameters*		
TNFα (pg/mL)	37.30 ± 13.68	Macrophages (% of area)		1.91 ± 0.4
TAOC (mmol/L trolox equivalent)	10.51 ± 0.29	Oxidative stress (% *vs.* age-matched ND)		100 ± 22.9
Lipids peroxidation (μmol/L TBARS)	20.6 ± 3.2	SOD activity (% inhibition rate)		0.97 ± 0.003
		Catalase activity (μmol/L)		0.272 ± 0.03
		Lipids peroxidation (μmol/L TBARS)		23 ± 0.8
M2	ND	HF	HFD	HFHF
*Plasmatic parameters*				
TNFα (pg/mL)	23.23 ± 7.82	13.63 ± 3.52	49.03 ± 20.06	13.84 ± 5.46
TAOC (mmol/L trolox equivalent)	7.64 ± 0.15	7.93 ± 0.16	7.58 ± 0.45	8.32 ± 0.25
Lipids peroxidation (μmol/L TBARS)	19.4 ± 2.0	41.9 ± 10.5	26.5 ± 5.3	43.3 ± 13.1
*Hepatic parameters*				
Macrophages (% of area)	4.34 ± 1.1	2.88 ± 1.2	2.58 ± 0.8	4.10 ± 2.1
Oxidative stress (% *vs.* age-matched ND)	100 ± 11.4	108.1 ± 17.7	168.2 ± 17.3*	151 ± 17.9*
SOD activity (% inhibition rate)	1.15 ± 0.13	0.99 ± 0.008	1.17 ± 0.09	1.08 ± 0.07
Catalase activity (μmol/L)	0.243 ± 0.03	0.187 ± 0.02	0.283 ± 0.04	0.257 ± 0.02
Lipids peroxidation (μmol/L TBARS)	20.4 ± 1.1	23.9 ± 0.1**	19.4 ± 0.7	18 ± 1.4
M8	ND	HF	HFD	HFHF
*Plasmatic parameters*				
TNFα (pg/mL)	25.63 ± 7.57	48.90 ± 9.74	97.98 ± 28.42*	130.7 ± 40.68***
TAOC (mmol/L trolox equivalent)	11.07 ± 0.08	11.24 ± 0.19	10.96 ± 0.09	11.10 ± 0.27
Lipids peroxidation (μmol/L TBARS)	20.1 ± 2.5	22.5 ± 3.3	38.8 ± 3.8**	48.0 ± 6.3*
*Hepatic parameters*				
Macrophages (% of area)	2.20 ± 1.5	2.37 ± 1.1	5.99 **±** 1.9	11.26 ± 2.4** ^**a**^
Oxidative stress (% *vs.* age-matched ND)	100 ± 6.3	110.2 ± 21.5	149.2 ± 15.3	206 ± 29.1** ^**a**^
SOD activity (% inhibition rate)	1.09 ± 0.04	0.94 ± 0.08	1.13 ± 0.14	1.17 ± 0.16
Catalase activity (μmol/L)	0.246 ± 0.03	0.120 ± 0.03**	0.207 ± 0.03	0.232 ± 0.01
Lipids peroxidation (μmol/L TBARS)	18 ± 1.2	15.7 ± 2.0	18.9 ± 2.1	16.5 ± 1.1

Effects of diet on plasmatic and hepatic inflammation and oxidative parameters during the study, at the beginning (M0) and after 2 (M2) and 8 months (M8) of normal diet (ND), normal diet + fructose (HF), high fat diet (HFD) and HFD + fructose (HFHF). In bold significant difference with age-matched ND rats and ^a^between HFD- and HFHF-rats. *p<0.05, **p<0.01, ***p<0.001

### HFHF and hepatic complications

**Fig. 2:**
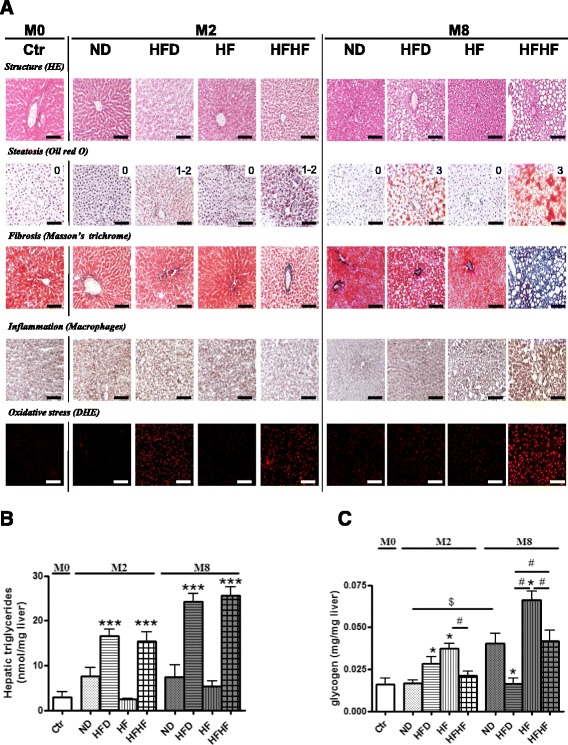
Evolution of hepatic complications. **a** Severity of hepatic complications was assessed by eosin/hematoxylin coloration and Oil-Red O for steatosis, Masson’s Trichrome for fibrosis, macrophages infiltration and oxidative stress by dihydroethidine (DHE) at the beginning (M0), after 2 (M2) and 8 (M8) months of normal diet (ND), normal diet + fructose (HF), high fat diet (HFD) and HFD + fructose (HFHF). The score of steatosis is noted under the picture (0, 1, 2, or 3). Results for 5 different experiments are shown. Bar scale = 100 μm. **b**-**c** Quantification of hepatic triglycerides (**b**) and glycogen (**c**) in frozen-liver tissue, at the beginning (M0), after 2 (M2) and 8 (M8) months of normal diet (ND), normal diet + fructose (HF), high fat diet (HFD) and HFD + fructose (HFHF). Results are shown as mean ± SEM of 5 different experiments. * Significant results *versus* age-matched ND-rats and # between two groups

HE analysis of the liver showed marked vacuolar degeneration in HFHF and HFD rats, indicating hepatic fat accumulation (Fig. 2a). Oil-red O-staining confirmed the accumulation of lipid droplets in HFHF and HFD rats. Steatosis increased between 2 and 8 months in HFHF and HFD rats (steatosis score: 1–2 to the maximum 3, according to Kleiner et al. [32]), associated with fibrosis and macrophage infiltration after 8 months only in HFHF rats. In fact, the HFHF diet was found to result in an increase in hepatic macrophages (*p* < 0.01) in comparison to HFD, HF and ND (Fig. 2a and Table 4). Biochemical analysis of the hepatic triglyceride content confirmed lipid accumulation, with a significant increase in triglycerides after 2 months, which was maintained after 8 months (Fig. 2b). The level of hepatic glycogen content was comparable in HFHF and ND rats during the study. HF was found to gradually increase glycogen levels from 2 to 8 months, whereas HFD transiently increased glycogen levels at 2 months (Fig. 2c).

HFHF induced a significant and persistent increase in hepatic ROS levels from 2 months and throughout the study in comparison to ND, without TBARS formation. HFD induced only a transient hepatic ROS formation after 2 months, not associated to TBARS complication. HF induced a transient increase of TBARS (*p* < 0.01) without ROS formation (Fig. 2a and Table 4). In spite of ROS observed in the liver, SOD and catalase activities were comparable in all groups during the study, excepted after 8 months in HF-rats where catalase activity was decreased (*p* < 0.01) (Table 4).

### HFHF and vascular function

**Fig. 3:**
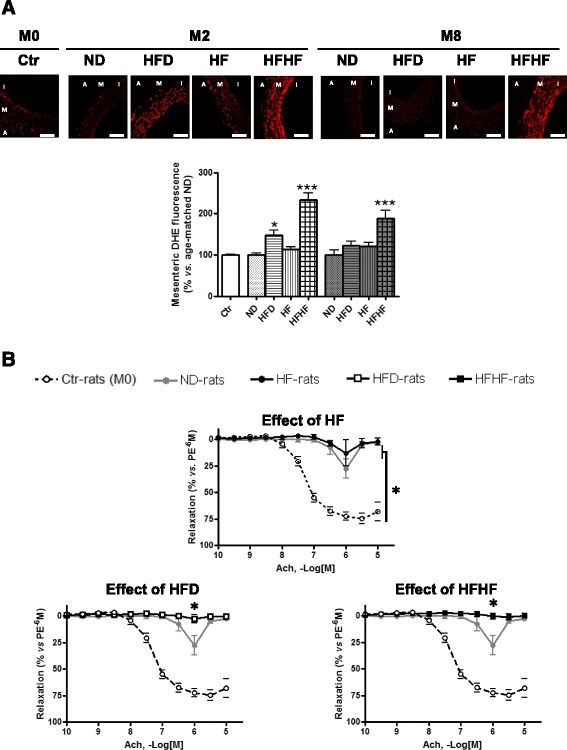
Evolution of vascular complications: oxidative stress and vascular reactivity of mesenteric artery. **a** *Higher panel.* Visualization of dihydroethidine (DHE) representative immunofluorescent staining of ROS in rats’ liver (**a**) and mesenteric artery (**b**) during the study, at the beginning (M0) and after 2 (2M) and 8 (8M) months of normal diet (ND), normal diet + fructose (HF), high fat diet (HFD) and HFD + fructose (HFHF). *Bottom panel.* Corresponding cumulative data. Results are shown as mean ± SEM of 5 different experiments. * Significant results *versus* age-matched ND-rats and # between HFD- and HFHF-rats. Bar scale = 100 μm. **b** Concentration–relaxation curves to acetylcholine (ACh) in mesenteric artery rings with endothelium from control-rats (M0), normal diet-rats (ND, ) after 8 months of diet and normal diet + fructose (HF, ●), high fat diet (HFD, □) and HFD + fructose (HFHF, ■). Experiments were performed in the presence of indomethacin (10^−5^ M) and N^ω^-nitro-l-arginine (L-NA, 10^−4^ M) to rule out the formation of vasoactive prostanoids and NO, respectively (EDHF-mediated component). Results are shown as means ± SEM of 5 different rats. *P < 0.05 represents a significant effect

The cumulative addition of acetylcholine caused concentration-dependent relaxations in isolated mesenteric arteries of Ctr-rats in the presence of indomethacin (10 μM) and *N*^*ω*^-nitro-L-arginine (100 μM) (Fig. 3). This EDHF-mediated component of relaxation is strongly decreased after 8 months in ND and HF rats and abolished in HFHF and HFD rats. The concentration-dependent relaxation induced by levcromakalim (0.1 nM–10 μM), an ATP-sensitive K^+^ channel opener, in mesenteric arteries from all groups, was maintained (respectively: relaxant effect at 10 μM, 94.5 ± 7.0 % in mesenteric arteries from Ctr-rats at M0 and at M8: 96.7 ± 0.9 % in ND-rats, 96 ± 2.2 % in HFHF-rats, 98 ± 0.7 % in HFD-rats and 103.5 ± 3.2 % in HF-rats, data not shown). Moreover, in the presence of indomethacin, *N*^*ω*^-nitro-L-arginine, and charybdotoxin plus apamin, acetylcholine-induced relaxations were abolished in all groups (data not shown).

Altogether, these results indicate that the EDHF-mediated component of relaxation is abolished by HFHF and HFD after 8 months, regardless of the age of rats.

Moreover, HFHF induced a significant and persistent increase in hepatic ROS levels from 2 months and throughout the study, in comparison to ND. HFD induced only a transient hepatic ROS formation after 2 months and HF had no effect on hepatic ROS (Fig. 2a and Table 4).

## Discussion

The present study demonstrates that only the combination of sugar and fat, present in beverage and diet (HFHF), allowed the development of T2D associated with long-term metabolic disorders such as maintenance of fasting hyperglycaemia, pre- and post-prandial hyperinsulinaemia, insulin resistance, glucose intolerance, and dyslipidaemia (hypertriglyceridaemia and hypercholesterolaemia). Moreover, HFHF was found to induce T2D-associated complications such as hepatic steatosis complicated by fibrosis, inflammation, and OS in the animals, regardless of their age. In addition, HFHF and HFD induced obesity associated with hyperleptinaeemia and endothelial dysfunction. However, HFD alone induced only transient insulin resistance, glucose intolerance, and hypertriglyceridaemia, without any impact on total cholesterol. In contrast, ND rats showed a decrease in insulin sensitivity, associated with glucose intolerance, with increasing age, thus minimizing the effects provided by the HFD. A transient metabolic disorders appears after 2 months of fructose beverage-intake only, characterized by a reduction of insulin sensitivity without abnormal weight gain, which may be due to an expansion of adipose cells [37] suggesting by hyperleptinaemia, even though total calorie intake (food and beverage, data not shown) did not differ between groups. These results are in accordance with the very few studies in humans reporting that the effects of fructose-rich diets, particularly on insulin sensitivity, appear to be dose-dependent [38, 39]. However, the dose of fructose administrated to rodents was higher (50–60 % of the diet) than that administered to humans (10–15 %).

Excessive consumption of fructose may affect the liver [40]. Indeed, this was found to increase the production of glycogen in HF and HFHF rats after 2 and 4 months. The conversion of glucose into glycogen is a key pathway by which the liver removes glucose from the portal vein after a meal, mediated by the bidirectional GLUT2 transporter and through gluconeogenesis [41]. Koo et al. [42] showed that liver glycogen is higher in HF-rats, indicating its conversion by gluconeogenesis. This increase in glycogen may represent a protective mechanism against fat accumulation in the liver [43], and against hyperglycaemia [44], as observed in the HF rats. However, after 8 months of HFHF, hepatic OS resulted in a decrease in the levels of glycogen, as reported by Castro MC et al. [45], throughout a NADPH oxidase pathway. Less significantly, after 2 months of HFD, the hepatic glycogen level was found to have increased through a second mechanism involving hyperinsulinaemia [40] and GSK-3 inactivation [46]. Therefore, only this mechanism is involved in glycogen increase in HFD rats, while both mechanisms contribute to increased glycogen levels in rats that were fed fructose.

In our study, only HFHF was found to induce the development and progression of steatosis into NASH. The factors causing progression of NAFLD to fibrosis and cirrhosis have not been defined in humans, in part because of the unavailability of human liver tissue for studies. Nevertheless, a series of mechanisms acting in synergy, or in stages, have been proposed to explain the transition and development of NAFLD to NASH. Factors that appear to be crucial involve increase in the vulnerability of steatotic hepatocytes, OS, alteration in the metabolism of lipids, mitochondrial dysfunction, and insulin resistance. The latter includes not only the metabolic consequences of insulin resistance and hyperinsulinaemia, but also changes in output of adipocytokines in fat tissue, which create a pro-inflammatory and potentially pro-fibrotic state [18, 47]. In fact, HFHF-induced NASH in this model is associated with fibrosis and macrophage infiltration, elevation of hepatic triglyceride level, and OS. Moreover the level of leptin, which also has pro-inflammatory properties and is considered an essential mediator of hepatic fibrosis [48], was significantly higher in HFHF than in HFD rats, despite the same weight gain for each group. This may highlight a body fat abnormality leading to higher hepatic complications [49]. These results are in accordance with the ‘two-hits’ hypothesis of Day and James [50], in which the accumulation of hepatic triglycerides constitutes the first hit of NASH pathogenesis, and OS followed by inflammation represents the second hit.

Hepatocytes have an elaborate system of enzymatic and non-enzymatic antioxidant defences to remove or neutralize ROS [51]. However, excessive levels of ROS may overwhelm the hepatocellular antioxidant defences, resulting in OS, hepatocyte injury, and cell death. In our model, HFHF and HFD rapidly induced hepatic OS, highlighting that OS as an important inducer of hepatic steatosis, whereas HF alone was neither associated with OS nor with steatosis. Short periods of hyperglycaemia are also able to induce OS [52], as observed after 2 months of HFD (higher but not significant fasting glycaemia), associated with steatosis. However, chronic hyperglycaemia, in addition to fluctuations in glucose level and hyperinsulinaemia (particularly in fasting conditions), leptin resistance, and inflammation exacerbate ROS formation and maintain OS [20]. ROS are understood to be important for the stimulating the production of type I collagen by hepatic stellate cells, therefore increasing extracellular matrix deposition during fibrogenesis [53]. These various disorders, which were observed in the HFHF-rats, but not the HF rats, after 8 months, explain the progression of NAFLD into NASH. Our results on liver complications are in accordance with the study of Rector et al. who demonstrated that mitochondrial dysfunction progressively contributes to the development of obesity-associated NAFLD in OLETF rats [54]. It can therefore be concluded that OS plays an important role in both the initial stages of steatosis, as well as its progression, especially in fibrosis and NASH.

Several studies have reported an association between NAFLD and cardiovascular disease-related complications (i.e. atherosclerosis) and hypothesise that common inflammatory and/or OS mediators are involved in inducing hepatic and peripheral vascular alterations [55]. In our study, EDHF-mediated relaxations were found to be blunted in the mesenteric artery of HFHF- and HFD-rats after 8 months of diet, but no significant similar effects were observed in rats fed the HF diet. Moreover, while ageing induced blunted NO-mediated relaxation, neither HF, HFD nor HFHF exacerbate this dysfunction (data not shown). So, NO-mediated relaxations were not affected by diets. These results are in accordance with the reduction of EDHF observed in other models of T2D (Goto-Katizaki rats, Zucker obese rats, and OLETF rats) [56], which do not show any effect on NO. However, studies of NO that report an increase, maintenance, or reduction in relaxation [56] in other types of arteries, species, and animals models may explain this variability. Another factor that influences endothelial function is the age of the animal. Indeed, previous studies have shown that aging is associated with a rapid (20 weeks) and progressive loss of endothelial function in rats [21]. In our study, after 8 months of treatment (at 10 months of age), ND rats developed endothelial dysfunction characterised by reduced NO- and EDHF-mediated relaxations in mesenteric arteries, without any effect on pathways involved in the relaxation of smooth muscle cells (data not shown). The same results were obtained for the aorta, where NO is the most important relaxant factor (data not shown). Therefore, in our study, only the EDHF component of relaxation was found to be blunted by HFHF and HFD after 8 months, in addition to age-induced endothelial dysfunction. Recent studies have reported that two factors are related to glucose-induced OS: the glucose variability and the glucose memory. Therefore, the endothelial dysfunction observed in HFD rats after 8 months may be explained in part by the long-lasting deleterious effects of glucose, which persist beyond the period of hyperglycaemia (observed only at 2 months), whereas HFHF rats have longer-lasting glycaemic fluctuation due to the persistence of glucose intolerance and hyperglycaemia in these rats. In addition, a number of disorders may induce endothelial dysfunction, such as obesity, visceral fat distribution [57], impaired fasting glucose, hyperglycaemia [58], insulin resistance [59], oxidative stress [60], and inflammation [61] all of which were observed in our models, and reported in other studies [62].

## Conclusions

Increased fat intake and Western diets have been linked to insulin resistance, impaired postprandial lipid metabolism, and the development or progression of NAFLD. However, data from recent animal experiments and human studies additionally implicate sugar consumption (primarily in the form of soft drinks, worldwide) in the development of diabetes mellitus and related metabolic diseases that raise cardiovascular risk. The latest WHO guidelines advise reducing sugar intake by 10 to 5 % of the daily calorie intake, in order to provide additional benefits. For many years, sugars (mainly fructose) have been added to processed foods, and our taste preferences are progressively shaped by more pronounced sweet flavours. Recent scientific reports identify fructose as the major player in the risk of developing T2D [62]. Therefore, fructose is an essential and indispensable element to consider for the development of an in vivo model of T2D that is most representative of the pathophysiology of the disease in humans. In our study, while the intake of fructose alone was found to accelerate metabolic disturbances associated with increasing age (insulin resistance and glucose intolerance), its association with HFD was found to result in the development of metabolic syndrome in T2D and liver and vascular complications. The administration of a combination of fructose and lipids was found to be ideal for the development of an experimentally induced T2D model. These different in vivo models (HF, HFD, and HFHF) should be useful as tools for testing candidates (natural or synthetic) in drug development for a broad spectrum of metabolic diseases.

## Abbreviations

DHE: dihydroethidine
EDHF: endothelium-derived-hyperpolarizing factor
HF: high-fructose beverages
HFD: high-fat diet
HFHF: high-fat-high fructose
IpGTT: intraperitoneal glucose tolerance
NAFLD: non-alcoholic fatty liver disease
NASH: non-alcoholic steatohepatitis
ND: normal diet
NO: nitric oxide
OS: oxidative stress
ROS: reactive oxygen species
T2D: type 2 diabetes
TAOC: total antioxidant capacity

## References

[1] Nolan CJ, Damm P, Prentki M (2011). Type 2 diabetes across generations: from pathophysiology to prevention and management. Lancet.

[2] Schwenk RW, Vogel H, Schurmann A (2013). Genetic and epigenetic control of metabolic health. Mol Metab.

[3] Dabelea D, Pettitt DJ (2001). Intrauterine diabetic environment confers risks for type 2 diabetes mellitus and obesity in the offspring, in addition to genetic susceptibility. J Pediatr Endocrinol Metab.

[4] Ludwig DS (2000). Dietary glycemic index and obesity. J Nutr.

[5] Tappy L, Le KA (2010). Metabolic effects of fructose and the worldwide increase in obesity. Physiol Rev.

[6] Elliott SS, Keim NL, Stern JS, Teff K, Havel PJ (2002). Fructose, weight gain, and the insulin resistance syndrome. Am J Clin Nutr.

[7] Ruxton CH, Gardner EJ, McNulty HM (2010). Is sugar consumption detrimental to health? A review of the evidence 1995–2006. Crit Rev Food Sci Nutr.

[8] Welsh JA, Sharma A, Abramson JL, Vaccarino V, Gillespie C, Vos MB (2010). Caloric sweetener consumption and dyslipidemia among US adults. JAMA.

[9] Dekker MJ, Su Q, Baker C, Rutledge AC, Adeli K (2010). Fructose: a highly lipogenic nutrient implicated in insulin resistance, hepatic steatosis, and the metabolic syndrome. Am J Physiol Endocrinol Metab.

[10] Vasdev S, Longerich L, Gill V (2004). Prevention of fructose-induced hypertension by dietary vitamins. Clin Biochem.

[11] Basu S, Yoffe P, Hills N, Lustig RH (2013). The relationship of sugar to population-level diabetes prevalence: an econometric analysis of repeated cross-sectional data. PLoS One.

[12] van Dam RM, Willett WC, Rimm EB, Stampfer MJ, Hu FB (2002). Dietary fat and meat intake in relation to risk of type 2 diabetes in men. Diabetes Care.

[13] RHaP D (1990). Ellenberg and Rifkin’s Diabetes Mellitus: Theory and Practice.

[14] Bugianesi E, Moscatiello S, Ciaravella MF, Marchesini G (2010). Insulin resistance in nonalcoholic fatty liver disease. Curr Pharm Des.

[15] Adams LA, Waters OR, Knuiman MW, Elliott RR, Olynyk JK (2009). NAFLD as a risk factor for the development of diabetes and the metabolic syndrome: an eleven-year follow-up study. Am J Gastroenterol.

[16] Targher G, Marra F, Marchesini G (2008). Increased risk of cardiovascular disease in non-alcoholic fatty liver disease: causal effect or epiphenomenon?. Diabetologia.

[17] Browning JD, Horton JD (2004). Molecular mediators of hepatic steatosis and liver injury. J Clin Invest.

[18] Takaki A, Kawai D, Yamamoto K (2013). Multiple hits, including oxidative stress, as pathogenesis and treatment target in non-alcoholic steatohepatitis (NASH). Int J Mol Sci.

[19] Droge W (2002). Free radicals in the physiological control of cell function. Physiol Rev.

[20] Valko M, Leibfritz D, Moncol J, Cronin MT, Mazur M, Telser J (2007). Free radicals and antioxidants in normal physiological functions and human disease. Int J Biochem Cell Biol.

[21] Dal-Ros S, Zoll J, Lang AL, Auger C, Keller N, Bronner C (2011). Chronic intake of red wine polyphenols by young rats prevents aging-induced endothelial dysfunction and decline in physical performance: role of NADPH oxidase. Biochem Biophys Res Commun.

[22] Bloch-Damti A, Bashan N (2005). Proposed mechanisms for the induction of insulin resistance by oxidative stress. Antioxid Redox Signal.

[23] Orasanu G, Plutzky J (2009). The pathologic continuum of diabetic vascular disease. J Am Coll Cardiol.

[24] Sena CM, Pereira AM, Seica R (2013). Endothelial dysfunction - a major mediator of diabetic vascular disease. Biochim Biophys Acta.

[25] Islam MS, du Loots T (2009). Experimental rodent models of type 2 diabetes: a review. Methods Find Exp Clin Pharmacol.

[26] Kim JH, Nishina PM, Naggert JK (1998). Genetic models for non insulin dependent diabetes mellitus in rodents. J Basic Clin Physiol Pharmacol.

[27] Liu RH, Mizuta M, Kurose T, Matsukura S (2002). Early events involved in the development of insulin resistance in Zucker fatty rat. Int J Obes Relat Metab Disord.

[28] Dourmashkin JT, Chang GQ, Gayles EC, Hill JO, Fried SK, Julien C (2005). Different forms of obesity as a function of diet composition. Int J Obes (Lond).

[29] Lenzen S (2008). The mechanisms of alloxan- and streptozotocin-induced diabetes. Diabetologia.

[30] Auberval N, Dal S, Bietiger W, Pinget M, Jeandidier N, Maillard-Pedracini E (2014). Metabolic and oxidative stress markers in Wistar rats after 2 months on a high-fat diet. Diabetol Metab Syndr.

[31] O’Rahilly S, Burnett MA, Smith RF, Darley JH, Turner RC (1987). Haemolysis affects insulin but not C-peptide immunoassay. Diabetologia.

[32] Shapiro A, Mu W, Roncal C, Cheng KY, Johnson RJ, Scarpace PJ (2008). Fructose-induced leptin resistance exacerbates weight gain in response to subsequent high-fat feeding. Am J Physiol Regul Integr Comp Physiol.

[33] Kleiner DE, Brunt EM, Van Natta M, Behling C, Contos MJ, Cummings OW (2005). Design and validation of a histological scoring system for nonalcoholic fatty liver disease. Hepatology.

[34] Dal S, Jeandidier N, Schaschkow A, Spizzo AH, Seyfritz E, Sookhareea C, et al. Portal or subcutaneous insulin infusion: efficacy and impact on liver inflammation. Fundamental & clinical pharmacology. 2015. 10.1111/fcp.12129 26095147

[35] Dal S, Jeandidier N, Seyfritz E, Bietiger W, Peronet C, Moreau F, et al. Oxidative stress status and liver tissue defenses in diabetic rats during intensive subcutaneous insulin therapy. Exp Biol Med. 2015. 10.1177/1535370215603837 PMC4935385 26385497

[36] Bradford MM (1976). A rapid and sensitive method for the quantitation of microgram quantities of protein utilizing the principle of protein-dye binding. Anal Biochem.

[37] Du L, Heaney AP (2012). Regulation of adipose differentiation by fructose and GluT5. Mol Endocrinol.

[38] Faeh D, Minehira K, Schwarz JM, Periasamy R, Park S, Tappy L (2005). Effect of fructose overfeeding and fish oil administration on hepatic de novo lipogenesis and insulin sensitivity in healthy men. Diabetes.

[39] Le KA, Faeh D, Stettler R, Ith M, Kreis R, Vermathen P (2006). A 4-wk high-fructose diet alters lipid metabolism without affecting insulin sensitivity or ectopic lipids in healthy humans. Am J Clin Nutr.

[40] Coate KC, Scott M, Farmer B, Moore MC, Smith M, Roop J (2010). Chronic consumption of a high-fat/high-fructose diet renders the liver incapable of net hepatic glucose uptake. Am J Physiol Endocrinol Metab.

[41] Feinman RD, Fine EJ (2013). Fructose in perspective. Nutr Metab.

[42] Koo HY, Wallig MA, Chung BH, Nara TY, Cho BH, Nakamura MT (2008). Dietary fructose induces a wide range of genes with distinct shift in carbohydrate and lipid metabolism in fed and fasted rat liver. Biochim Biophys Acta.

[43] Lopez-Soldado I, Zafra D, Duran J, Adrover A, Calbo J, Guinovart JJ (2015). Liver glycogen reduces food intake and attenuates obesity in a high-fat diet-fed mouse model. Diabetes.

[44] Ros S, Garcia-Rocha M, Calbo J, Guinovart JJ (2011). Restoration of hepatic glycogen deposition reduces hyperglycaemia, hyperphagia and gluconeogenic enzymes in a streptozotocin-induced model of diabetes in rats. Diabetologia.

[45] Castro MC, Francini F, Schinella G, Caldiz CI, Zubiria MG, Gagliardino JJ (2012). Apocynin administration prevents the changes induced by a fructose-rich diet on rat liver metabolism and the antioxidant system. Clin Sci.

[46] Farese RV, Sajan MP, Standaert ML (2005). Insulin-sensitive protein kinases (atypical protein kinase C and protein kinase B/Akt): actions and defects in obesity and type II diabetes. Exp Biol Med.

[47] Sutti S, Jindal A, Locatelli I, Vacchiano M, Gigliotti L, Bozzola C (2014). Adaptive immune responses triggered by oxidative stress contribute to hepatic inflammation in NASH. Hepatology.

[48] Tsochatzis E, Papatheodoridis GV, Archimandritis AJ (2006). The evolving role of leptin and adiponectin in chronic liver diseases. Am J Gastroenterol.

[49] Lee UE, Friedman SL (2011). Mechanisms of hepatic fibrogenesis. Best Pract Res Clin Gastroenterol.

[50] Day CP, James OF (1998). Steatohepatitis: a tale of two “hits”?. Gastroenterology.

[51] Masayasu I, Arias IM, Boyer JL, Fausto, Jakoby WB, Schachter DA, Shafritz DA (1994). Protective mechanism against reactive oxygen species. The liver: Biology and Pathobiology.

[52] Monnier L, Mas E, Ginet C, Michel F, Villon L, Cristol JP (2006). Activation of oxidative stress by acute glucose fluctuations compared with sustained chronic hyperglycemia in patients with type 2 diabetes. JAMA.

[53] Nieto N, Greenwel P, Friedman SL, Zhang F, Dannenberg AJ, Cederbaum AI (2000). Ethanol and arachidonic acid increase alpha 2(I) collagen expression in rat hepatic stellate cells overexpressing cytochrome P450 2E1. Role of H2O2 and cyclooxygenase-2. J Biol Chem.

[54] Rector RS, Thyfault JP, Uptergrove GM, Morris EM, Naples SP, Borengasser SJ (2010). Mitochondrial dysfunction precedes insulin resistance and hepatic steatosis and contributes to the natural history of non-alcoholic fatty liver disease in an obese rodent model. J Hepatol.

[55] Targher G, Chonchol M, Miele L, Zoppini G, Pichiri I, Muggeo M (2009). Nonalcoholic fatty liver disease as a contributor to hypercoagulation and thrombophilia in the metabolic syndrome. Semin Thromb Hemost.

[56] Shi Y, Vanhoutte PM (2009). Reactive oxygen-derived free radicals are key to the endothelial dysfunction of diabetes. J Diabetes.

[57] Arcaro G, Zamboni M, Rossi L, Turcato E, Covi G, Armellini F (1999). Body fat distribution predicts the degree of endothelial dysfunction in uncomplicated obesity. Int J Obes Relat Metab Disord.

[58] Vehkavaara S, Seppala-Lindroos A, Westerbacka J, Groop PH, Yki-Jarvinen H (1999). In vivo endothelial dysfunction characterizes patients with impaired fasting glucose. Diabetes Care.

[59] Steinberg HO, Chaker H, Leaming R, Johnson A, Brechtel G, Baron AD (1996). Obesity/insulin resistance is associated with endothelial dysfunction. Implications for the syndrome of insulin resistance. J Clin Invest.

[60] Griendling KK, FitzGerald GA (2003). Oxidative stress and cardiovascular injury: Part II: animal and human studies. Circulation.

[61] Cleland SJ, Sattar N, Petrie JR, Forouhi NG, Elliott HL, Connell JM (2000). Endothelial dysfunction as a possible link between C-reactive protein levels and cardiovascular disease. Clin Sci.

[62] Zhang H, Dellsperger KC, Zhang C (2012). The link between metabolic abnormalities and endothelial dysfunction in type 2 diabetes: an update. Basic Res Cardiol.

